# Reconstruction and Comparison of the Metabolic Potential of Cyanobacteria *Cyanothece* sp. ATCC 51142 and *Synechocystis* sp. PCC 6803

**DOI:** 10.1371/journal.pone.0048285

**Published:** 2012-10-31

**Authors:** Rajib Saha, Alex T. Verseput, Bertram M. Berla, Thomas J. Mueller, Himadri B. Pakrasi, Costas D. Maranas

**Affiliations:** 1 Department of Chemical Engineering, The Pennsylvania State University, University Park, Pennsylvania, United States of America; 2 Department of Energy, Environmental, and Chemical Engineering, Washington University, St. Louis, Missouri, United States of America; 3 Department of Biology, Washington University, St. Louis, Missouri, United States of America; Hospital for Sick Children, Canada

## Abstract

Cyanobacteria are an important group of photoautotrophic organisms that can synthesize valuable bio-products by harnessing solar energy. They are endowed with high photosynthetic efficiencies and diverse metabolic capabilities that confer the ability to convert solar energy into a variety of biofuels and their precursors. However, less well studied are the similarities and differences in metabolism of different species of cyanobacteria as they pertain to their suitability as microbial production chassis. Here we assemble, update and compare genome-scale models (*i*Cyt773 and *i*Syn731) for two phylogenetically related cyanobacterial species, namely *Cyanothece* sp. ATCC 51142 and *Synechocystis* sp. PCC 6803. All reactions are elementally and charge balanced and localized into four different intracellular compartments (i.e., periplasm, cytosol, carboxysome and thylakoid lumen) and biomass descriptions are derived based on experimental measurements. Newly added reactions absent in earlier models (266 and 322, respectively) span most metabolic pathways with an emphasis on lipid biosynthesis. All thermodynamically infeasible loops are identified and eliminated from both models. Comparisons of model predictions against gene essentiality data reveal a specificity of 0.94 (94/100) and a sensitivity of 1 (19/19) for the *Synechocystis i*Syn731 model. The diurnal rhythm of *Cyanothece* 51142 metabolism is modeled by constructing separate (light/dark) biomass equations and introducing regulatory restrictions over light and dark phases. Specific metabolic pathway differences between the two cyanobacteria alluding to different bio-production potentials are reflected in both models.

## Introduction

Cyanobacteria represent a widespread group of photosynthetic prokaryotes [1]. By contributing oxygen to the atmosphere, they played an important role in the precambrian phase [2]. Cyanobacteria are primary producers in aquatic environments and contribute significantly to biological carbon sequestration, O_2_ production and the nitrogen cycle [3]–[5]. Their inherent photosynthetic capability and ease in genetic modifications are two significant advantages over other microbes in the industrial production of valuable bioproducts [6]. In contrast to other microbial production processes requiring regionally limited cellulosic feedstocks, cyanobacteria only need CO_2_, sunlight, water and a few mineral nutrients to grow [6]. Sunlight is the most abundant source of energy on earth. The incident solar flux onto the USA alone is approximately 23,000 terawatts which dwarfs the global energy usage of 3.16 terawatts [7]. Cyanobacteria perform photosynthesis more efficiently than terrestrial plants (3–9% vs. 2.4–3.7%) [8]. The short life cycle and transformability of cyanobacteria combined with a detailed understanding of their biochemical pathways are significant advantages of cyanobacteria as efficient platforms for harvesting solar energy and producing bio-products such as short chain alcohols, hydrogen and alkanes [6].

The genus *Cyanothece* includes unicellular cyanobacteria that can fix atmospheric nitrogen. *Cyanothece* sp. ATCC 51142 (hereafter *Cyanothece* 51142) is one of the most potent diazotrophs characterized and the first to be completely sequenced [9]. Studies show that it can fix atmospheric nitrogen at rates higher than many filamentous cyanobacteria and also accommodate the biochemically incompatible processes of photosynthesis and nitrogen fixation within the same cell by temporally separating them [10]. *Synechocystis* sp. PCC 6803 (hereafter *Synechocystis* 6803), the first photosynthetic organism with a completely sequenced genome [11], is probably the most extensively studied model organism for photosynthetic processes [12]. It is also closely related to *Cyanothece* 51142 and shares many characteristics with all *Cyanothece* [9]. The genome of *Cyanothece* 51142 is about 35% larger than that of *Synechocystis* 6803 mostly due to the presence of nitrogen fixation and temporal regulation related genes in *Cyanothece* 51142 [9]. *Synechocystis* 6803 has been the subject of many targeted genetic manipulations (e.g., expression of heterologous gene products) as a photo-biological platform for the production of valuable chemicals such as poly-beta-hydroxybutyrate, isoprene, hydrogen and biofuels [12]–[20]. However, genetic tools for *Cyanothece* 51142 are still lacking thus hampering its wide use as a bio-production strain even though it has many attractive native pathways. For example, *Cyanothece* 51142 can produce (in small amounts) pentadecane and other hydrocarbons while containing a novel (though incomplete) non-fermentative pathway for producing butanol [21], [22].

A breakthrough in solar biofuel production will require following one of two strategies: 1) obtaining photosynthetic strains that naturally have high-throughput pathways analogous to those in known biofuel producers, or 2) creating cellular environments conducive for heterologous enzyme function. Despite its attractive capabilities including nitrogen fixation and H_2_ production [19], unfortunately genetic tools are not currently available to efficiently test engineering interventions directly for *Cyanothece* 51142. Therefore, a promising path forward may be to use *Synechocystis* 6803 as a “proxy” (for which a comprehensive genetic toolkit is available) and subsequently transfer knowledge gained during experimentation with *Synechocystis* 6803 to *Cyanothece* 51142. This requires high quality metabolic models for both organisms. Comprehensive genome-wide metabolic reconstructions include the complete inventory of metabolic transformations of a given cyanobacterial system. Comparison of the metabolic capabilities of *Cyanothece* 51142 and *Synechocystis* 6803 derived from their corresponding genome-scale models will provide valuable insights into their niche biological functions and also open up new avenues for economical biofuel production.

Genome-scale models (GSM) contain gene to protein to reaction associations (GPRs) along with a stoichiometric representation of all possible biotransformations known to occur in an organism combined with a set of appropriate regulatory constraints on each reaction flux [23], [24]. By defining the global metabolic space and flux distribution potential, GSMs can assess allowable cellular phenotypes under specific environmental conditions [23], [24]. The first genome-scale model for *Cyanothece* 51142 was recently published [25]. The authors addressed the complexity of the electron transport chain (ETC) and explored further the specific roles of photosystem I (PSI) and photosystem II (PSII). In contrast, *Synechocystis* 6803 has been the target for metabolic model reconstruction for quite some time [12], [26]–[32]. Most of these earlier efforts for *Synechocystis* 6803 focused on only central metabolism [26]–[28]. Knoop *et al.* [12] and Montagud *et al.* [30], [31] developed genome-scale models for *Synechocystis* 6803, analyzed growth under different conditions, identified gene knock-out candidates for enhanced succinate production and performed flux coupling analysis to detect potential bottlenecks in ethanol and hydrogen production. A more recent model describes in detail the photosynthetic apparatus, identifies alternate electron flow pathways and highlights the high photosynthetic robustness of *Synechocystis* 6803 during photoautotrophic metabolism [32]. All these efforts have brought about an improved understanding of the metabolic capabilities of *Synechocystis* 6803 and cyanobacterial systems in general.

This paper introduces high-quality genome-scale models for *Cyanothece* 51142 *i*Cyt773 and *Synechocystis* 6803 *i*Syn731 (as shown in Table 1) that integrate all recent developments [25], [32], supplements them with additional literature evidence and highlights their similarities and differences (see Files S1, S2, S7 and S8 for detailed description of these models). As many as 322 unique reactions are introduced in the *Synechocystis i*Syn731 model and 266 in *Cyanothece i*Cyt773. New pathways include, among many, a TCA bypass [33], heptadecane biosynthesis [21] and detailed fatty acid biosynthesis in *i*Syn731 and comprehensive lipid and pigment biosynthesis and pentadecane biosynthesis [21] in *i*Cyt773. For the first time, not only extensive gene essentiality data [34] is used to assess the quality of the developed model (i.e., *i*Syn731) but also the allowable model metabolic phenotypes are contrasted against MFA flux data [35]. The diurnal rhythm of *Cyanothece* metabolism is modeled for the first time via developing separate (light/dark) biomass equations and regulating metabolic fluxes based on available protein expression data over light and dark phases [36].

**Table 1:** *Synechocystis* 6803 *i*Syn731 and *Cyanothece* 51142 *i*Cyt773 model statistics.

	*Synechocystis 6803 i*Syn731 model	*Cyanothece 51142 i*Cyt773 model
**Included genes**	731	773
**Proteins**	511	465
Single functional proteins	348	336
Multifunctional proteins	91	83
Isozymes	4	1
Multimeric proteins	32	22
Others^a^	36	23
**Reactions**	1,156	946
Metabolic reactions	972	761
Transport reactions	127	128
*GPR associations*		
Gene associated (metabolic/transport)	827	686
Spontaneous^b^	180	158
Nongene associated (metabolic/transport)	59	16
No protein associated	90	86
Exchange reactions	57	57
**Metabolites** c	996	811
Cytosolic	862	675
Carboxisomic	8	8
Thylakoidic	10	9
Periplasmic	59	62
Extracellular	57	57

a Others include proteins involve in complex relationships, e.g. multiple proteins act as protein complex which is one of the isozymes for any specific reaction.

b Spontaneous reactions are those without any enzyme as well as gene association.

c Metabolites represent total number of metabolites with considering their compartmental specificity.

## Materials and Methods

### Measurement of Biomass Precursors

#### Growth conditions

Wild-type *Synechocystis* 6803 and *Cyanothece* 51142 were grown for several days from an initial OD_730_ of ∼0.05 to ∼0.4. *Synechocystis* 6803 was grown in BG-11 medium [92] and *Cyanothece* 51142 in ASP2 medium [93] with (+N) or without (−N) nitrate. All cultures were grown in shake flasks with continuous illumination of ∼100 µmol photons/m^2^/sec provided from cool white fluorescent tubes. *Synechocystis* was maintained at 30°C and *Cyanothece* at 25°C. For *Synechocystis,* the illumination was constant and doubling time was ∼24 hours. *Cyanothece* alternated between 12 hours of light and 12 hours of darkness, with a doubling time of ∼48 hours.

#### Pigments

1 mL of cells of both *Synechocystis* 6803 and *Cyanothece* 51142 (from light and dark phases) was pelleted and extracted twice with 5 mL 80% aqueous acetone and the extracts pooled. Spectra of this extract and of a sample of whole cells were taken on a DW2000 spectrophotometer (Olis, GA, USA) against 80% acetone or BG-11 media as a reference. Chlorophyll a contents were calculated as reported [94] from the acetone extract. Total carotenoid concentrations were also calculated from the acetone extract according to a published method [95]. The relative amounts of different carotenoids included in the biomass equation were estimated according to known ratios [96]. Concentrations of phycocyanin were estimated from the spectra of intact cells [97]. All measurements were taken in triplicate.

#### Amino acids

Total protein contents were measured using a Pierce BCA Assay kit. Amino acid proportions were determined according to published shotgun proteomics data for both *Cyanothece* 51142 and *Synechocystis* 6803 across a range of conditions [84] according to the following procedure: From peptide-level data, each mass spectral observation of a peptide was taken as an instance of a particular protein. The amino acid composition of each protein was taken from data in Cyanobase (http://genome.kazusa.or.jp/cyanobase) and thus the ‘proteome’ was taken to include all of the proteins whose peptides were observed in our data set, in proportion according to how often their peptides were observed. Amino acid frequencies were averaged across the proteome by a weighting factor of number of observations divided by the number of amino acids in the protein, similar to RPKM normalization for next-gen sequencing [98].

#### Other cellular components

The compositions of other cellular components of *Synechocystis* 6803 and *Cyanothece* 51142 were estimated based on values in the literature. DNA and RNA contents for *Synechocystis* 6803 were reported by Shastri and Morgan [27]. The remaining biomass components of *Synechocystis* 6803 (i.e., lipid, soluble pool and inorganic ions) were extracted from the measurements carried out by Nogales et al. [32]. For *Cyanothece* 51142, biochemical compositions of macromolecules such as lipids, RNA, DNA and soluble pool were extracted from the measurements reported by Vu et al. [25].

### Model Simulations

Flux balance analysis (FBA) [99] was employed in both the model validation and model testing phases. *Cyanothece i*Cyt773 and *Synechocystis i*Syn731 models were evaluated in terms of biomass production under several scenarios: light and dark phases, heterotrophic and mixotrophic conditions. Flux distributions for each one of these states were inferred using FBA:

Maximize 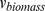


Subject to
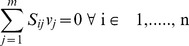
(1)

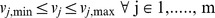
(2)


Here, *S_ij_* is the stoichiometric coefficient of metabolite *i* in reaction *j* and *v_j_* is the flux value of reaction *j*. Parameters *v_j,min_* and *v_j,max_* denote the minimum and maximum allowable fluxes for reaction *j,* respectively. Light and dark phases in *Cyanothece* 51142 are represented via modifying the minimum or maximum allowable fluxes with the following constraints, respectively:
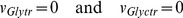
(3)

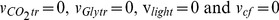
(4)


Here, *v_Biomass_* is the flux of biomass reaction and *v_Glytr_*, *v_Glyctr_* and *v_CO2tr_* are the fluxes of glycerol, glycogen and carbon dioxide transport reactions and *v_light_* and *v_cf_* are the fluxes of light reactions and carbon fixation reactions. For light phase, constraint (3) was included in the linear model, whereas for dark phase constraint (4) was included.

Once the *Synechocystis i*Syn731 model was validated, it was further tested for *in silico* gene essentiality. The following constraint(s) was included individually in the linear model to represent any mutant:
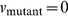
(5)


Here, *v_mutant_* represents flux of reaction(s) associated with any genetic mutation.

Flux variability analysis [100] for the reactions (for which photoautotrophic ^13^C MFA measurements [35] were available) was performed based on the following formulation:

Maximize/Minimize 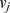


Subject to
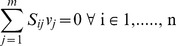
(6)

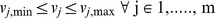
(7)

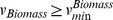
(8)


Here, 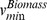 is the minimum level of biomass production. In this case we fixed it to be the optimal value obtained under light condition for the *Synechocystis i*Syn731 model.

CPLEX solver (version 12.1, IBM ILOG) was used in the GAMS (version 23.3.3, GAMS Development Corporation) environment for implementing GapFind and GapFill [39] and solving the aforementioned optimization models. All computations were carried out on Intel Xeon E5450 Quad-Core 3.0 GH and Intel Xeon E5472 Quad-Core 3.0 GH processors that are the part of the lionxj cluster (Intel Xeon E type processors and 96 GB memory) of High Performance Computing Group of The Pennsylvania State University.

## Results and Discussion

### Model Components

#### Biomass composition and diurnal cycle

The biomass equation approximates the dry biomass composition by draining all building blocks or precursor molecules in their physiologically relevant ratios. Most of the earlier genome-scale modeling efforts [12], [29], [30] of *Synechocystis* 6803 contain approximate biomass equations completely or partially adopted from other species without direct measurements. This can adversely affect the accuracy of maximum biomass yield calculations, gene essentiality predictions and knockouts for overproduction.

Biomass composition for *Synechocystis i*Syn731 and *Cyanothece i*Cyt773 models were generated by defining all essential cellular biomass content values by experimental measurement or collection from existing literature (see ‘Materials and Methods’ for detail). Macromolecules present in both cyanobacteria such as protein, carbohydrates, lipids, DNA, RNA, pigments, soluble pool and inorganic ions were assigned to their corresponding metabolic precursors (e.g., L-glycine, glucose, 16C-lipid, ATP, dGTP, beta-carotene, coenzyme A and potassium respectively) (see File S3 for the complete list of biomass components). Based on the experimental measurements of precursor molecules needed to form a gram of the biomass, stoichiometric coefficients were assigned. For *Synechocystis* 6803 we measured compositions of proteins and pigments and extracted compositions of the remaining biomass macromolecules from the model by Nogales *et al.* [32]. Thereby we developed biomass equations for three different conditions: photoautotrophic, mixotrophic and heterotrophic (see File S3). Experimental measurements (described in the Materials and Methods section and also in File S3) showed that biomass composition (i.e., mainly pigments) varies for *Cyanothece* 51142 between light and dark conditions and nitrogen supplementation. Since pigments such as chlorophyll, carotenoids and phycocyanobilin play important roles in photosynthetic processes their quantities are consequently higher under light conditions. In the presence of light *Cyanothece* 51142 uses photosynthesis to store solar energy in the form of carbohydrates (i.e., glycogen), while in dark it expends that energy to fix nitrogen. Surprisingly, no significant change was measured in the carbohydrate pool between light and dark phases due to infinitesimal contribution of photosynthetically stored carbohydrates to total carbohydrate content in the biomass of *Cyanothece* 51142. Aggregate quantities of the remaining biomass macromolecules for *Cyanothece* 51142 such as lipids, RNA, DNA and soluble pool were extracted from the most recent *Cyanothece* 51142 model by Vu *et al.* [25] to develop biomass equations for light and dark phases (File S3).

An earlier characterization study for *Cyanothece* 51142 revealed that 113 proteins are expressed in higher abundance in the light phase while 137 are expressed in higher abundance in dark conditions [36]. The constructed model spans 26 light-specific proteins, associated with 36 reactions mainly involved in fatty acid, pigment, and amino acid metabolism and 11 dark-specific proteins accounting for 16 reactions from glycolysis, purine, pyrimidine, pyruvate, and amino acid metabolism (Files S4). Separate biomass equations as well as two regulatory structures for the model were derived in order to represent diurnal metabolic differences for *Cyanothece* 51142 (File S4). In contrast, diurnal differences observed in *Synechocystis* 6803 [37] are less pronounced (i.e., observed for only 54 genes) and less well functionally annotated (i.e., 32 genes with *‘unassigned’* functions). When compared to existing biomass equations of *Synechocystis* 6803 [12], [30] we found significantly lower values for the percent weight contribution of proteins towards the biomass pool (i.e., 52% for *Synechocystis* 6803 and 53% for *Cyanothece* 51142 vs. 84% [12] and 66% [30], respectively). The new protein biomass contribution is in better agreement with the previously reported value of 55% for *Cyanothece* 51142 [38].

#### Identification and correction of network gaps

Upon ensuring biomass formation, GapFind [39] was applied to assess network connectivity and blocked metabolites. By applying Gapfill [39] putative reconnection hypotheses were identified for blocked metabolites. Only the suggested modifications that were independently corroborated using literature sources and also did not lead to the introduction of thermodynamically infeasible cycle were included in the model. For *Synechocystis i*Syn731 model, GapFind [39] identified 207 blocked metabolites. Note that there exist 125 blocked metabolites in the *i*JN678 model [32]. GapFill [39] identified unblocking hypotheses for 138 blocked metabolites. However, 88 of them led to the generation of infeasible thermodynamic cycles and thus were excluded. For only 5 blocked metabolites corroborating evidence for reconnection was obtained by adding 10 reactions (i.e., 2 metabolic, 4 transport and 4 exchange reactions). The added metabolic reactions have unknown gene associations (see File S1 for detailed information) while all 4 added transport reactions involve passive diffusion and thus are not associated with any specific gene(s) or protein(s). Ultimately, the 45 remaining blocked metabolites with GapFill suggested (but unconfirmed) reconnection mechanisms along with 69 blocked metabolites with no reconnection hypotheses were retained in the model *i*Syn731, while metabolites such as ubiquinone, which was proposed as an alternate substrate for succinate dehydrogenase [32] was excluded from *i*Syn731.

For the *Cyanothece i*Cyt773 model, 74 blocked metabolites were found after applying GapFind [39]. Note that there are 66 blocked metabolites in *i*Cce806 [25]. Two exchange reactions were added to allow the uptake of glucose and thyaminose ensuring biomass production under heterotrophic or mixotrophic conditions. Four blocked metabolites directly adopted from *i*Cce806 (during the draft model creation phase) were linked to five reactions with spurious gene associations and thus both metabolites and reactions were removed from *i*Cyt773. GapFill [39] suggested re-connection mechanisms for 52 blocked metaboloites (out of a total of 70). However, for 12 blocked metabolites the re-connection model modifications led to the creation of thermodynamically infeasible cycles and thus were discarded. Corroborating evidence for the reconnection of 30 blocked metabolites was identified through the addition of 19 GapFill suggested reactions (i.e., 8 metabolic, 7 transport and 4 exchange reactions). Of the eight added metabolic reactions we found direct literature evidence for five, homology-based evidence for one while two reactions are spontaneous (see File S2 for detailed information). All seven added transport reactions are through passive diffusion and thus are not connected with any specific gene(s) or protein(s). Ten remaining blocked metabolites with GapFill suggested reconnection hypotheses (along with 22 with no reconnection hypotheses) were left blocked in *i*Cyt773 as no information to corroborate the GapFill suggested changes was found in the published literature and databases. For example, biotin is produced in *Cyanothece* 51142; however, there is no literature evidence to support the presence of the initial step of the primary production pathway (i.e., conversion of pimeloyl-CoA from pimelate) and the intermediate step (i.e., biotransformation of 7,8-diamino-nonanoate from 8-amino-7-oxononanoate). This indicates that *Cyanothece* 51142 may utilize a currently unknown pathway for producing biotin. The six other blocked metabolites are involved in the nonfermentative alcohol production pathway (as explained in model comparison section) known to be incomplete in *Cyanothece* 51142. Table 2 summarizes the results related to connectivity restoration of *Synechocystis i*Syn731 and *Cyanothece i*Cyt773 models.

**Table 2:** Summary of connectivity restoration in *Synechocystis* 6803 *i*Syn731 and *Cyanothece* 51142 *i*Cyt773 models.

	*Synechocystis 6803 i*Syn731	*Cyanothece 51142 i*Cyt773
**Number of blocked metabolites**	207	74
**Number of metabolites with GapFill ** [**39**] ** suggested reconnection strategies**	138	52
**Number of metabolites whose reconnection forms a cycle**	88	12
**Number of metabolites with validated reconnection mechanisms**	5	30
**Number of added reactions to the model**	10	19

#### GPR associations and elemental and charge balancing

GPR associations connect genotype to phenotype by linking gene(s) that code for the protein(s) that catalyze a particular reaction. They are important to trace correctly as they provide the means to target at the gene level any change in the network desired at the reaction level. This is critical because genes may catalyze multiple reactions in multiple pathways. Many earlier models for *Synechocystis* 6803 do not provide in detail complex GPR associations, rather list only gene(s) and enzyme(s) involved in a specific reaction [12], [30], [31]. For both *i*Cyt773 and *i*Syn731 models, we included comprehensive GPR associations (see Table 1 for detail information). All four intracellular compartments (i.e., periplasm, cytosol, thylakoid lumen and carboxysome) were assumed to have the same pH (7.2) and subsequently, metabolites were assigned appropriate protonation states corresponding to this pH and each reaction was elementally and charge balanced.

Under high light intensity in photoautotrophic conditions, *Cyanothece i*Cyt773 model produces 0.026 mole biomass/mole carbon fixed whereas *Synechocystis i*Syn731 yields 0.021 mole biomass/mole carbon fixed. These yields are almost identical to the ones calculated using the most recent models of *Cyanothece* 51142 [25] and *Synechocystis* 6803 [32]. Experimental measurements of biomass yields are in the same order of magnitude with model predictions for the two organisms (i.e., 0.072 [25], [40] and 0.082 mole biomass/mole carbon fixed [41]), respectively.

### Comparison of *i*Syn731 Model Predicted Flux Ranges Against Experimental Measurements

**Figure 1:**
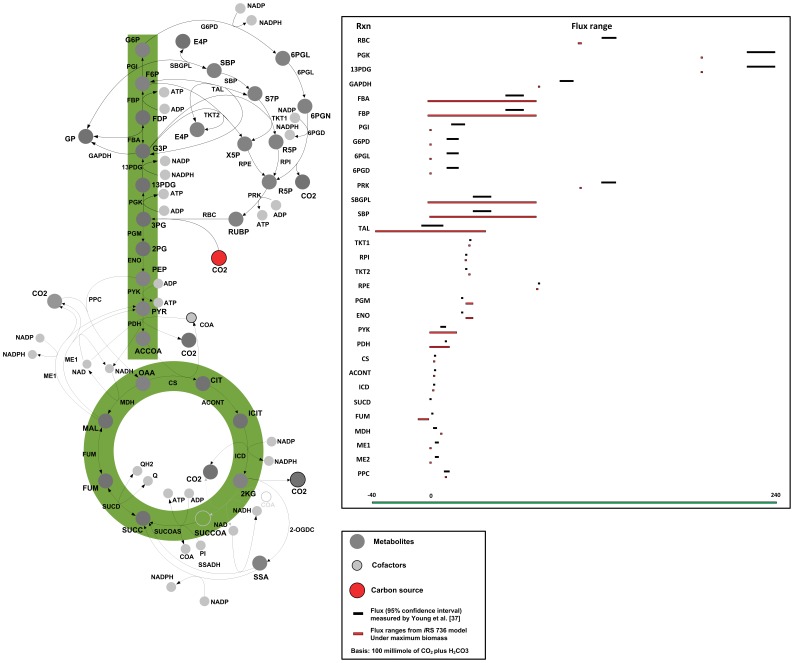
Comparison of model derived and experimentally measured [**35**] flux ranges for *Synechocystis* 6803 under the maximum biomass condition. Basis is 100 millimole of CO_2_ plus H_2_CO_3_.

We superimposed photoautotrophic flux measurements [35] for *Synechocystis* 6803 onto *i*Syn731 model to assess if the measurements are consistent with the model and whether the biomass maximization assumption correctly apportions fluxes to the metabolic network. For each reaction that was assigned a flux we calculated the flux-range under the maximum biomass assumption. Table 3 and Figure 1 summarize the obtained results for a basis of 100 millimole of CO_2_ plus H_2_CO_3_ uptake [35]. In seven (out of thirty one) cases the measured flux is fully contained within the model predicted ranges obtained upon maximizing biomass formation implying model consistency with MFA measurements. In contrast, under the maximum biomass assumption for thirteen fluxes the ranges underestimate and for four fluxes the ranges overestimate the experimentally deduced flux ranges while for seven fluxes the model derived flux ranges partially overlap with the experimental ones.

**Table 3:** Comparison of ^13^C MFA flux measurements [35] vs. model-predicted flux ranges.

Reaction	Flux measurements by Young *et al.*, 2011 [35]		Flux ranges predicted by *i*JN678 model *(With max biomass)*		Flux ranges predicted by *i*Syn731 model *(With max biomass)*	
	95% LB	95% UB	LB	UB	LB	UB
**RBC**	123.00	132.00	109.02	109.10	102.49	106.33
**PGK**	219.00	237.00	187.11	187.25	182.70	182.92
**13PDG**	219.00	237.00	187.11	196.36	182.70	201.96
**GAPDH**	90.00	99.00	74.98	75.07	73.40	73.50
**FBA**	53.00	66.00	−0.17	74.85	−0.08	73.17
**FBP**	53.00	66.00	0.00	74.85	0.00	73.17
**PGI**	15.00	24.00	0.68	0.73	0.82	0.84
**G6PD**	12.00	21.00	0.00	0.05	0.00	0.03
**6PGL**	12.00	21.00	0.00	0.05	0.00	0.03
**6PGD**	12.00	21.00	0.00	0.05	0.00	0.03
**PRK**	123.00	132.00	109.02	109.10	106.24	106.32
**SBGPL**	29.00	43.00	−0.17	74.85	−0.08	73.17
**SBP**	29.00	43.00	0.00	74.85	0.00	73.17
**TAL**	−6.00	9.00	−36.74	38.28	−35.93	37.32
**TKT1**	37.20	37.50	36.57	36.60	36.66	36.79
**RPI**	35.40	35.70	35.18	35.21	35.82	35.86
**TKT2**	35.40	35.70	37.25	37.28	36.18	36.23
**RPE**	75.50	76.20	73.83	73.88	72.01	72.10
**PGM**	22.90	23.60	26.83	26.95	25.92	29.79
**ENO**	23.40	23.80	26.84	26.95	25.92	29.79
**PYK**	7.90	11.10	0.00	13.88	0.00	16.72
**PDH**	11.50	12.00	0.00	8.97	0.00	13.46
**CS**	3.00	3.40	2.15	2.21	1.35	1.37
**ACONT**	3.00	3.40	2.15	2.21	1.35	1.37
**ICD**	3.00	3.00	2.15	2.21	1.32	1.37
**SUCD**	0.00	0.40	0.00	0.00	0.00	0.00
**FUM**	1.70	2.00	−5.44	1.55	−7.26	1.49
**MDH**	1.90	5.20	5.35	5.61	7.15	7.32
**ME1**	3.70	6.90	0.00	0.17	0.00	0.08
**ME2**	3.70	6.90	−	−	0.00	0.08
**PPC**	9.90	13.20	11.74	11.98	12.25	12.37

Perhaps the most informative discrepancy is for the CO_2_ fixing RuBisCO (RBC) reaction, which has a measured flux range of (123.00 to 132.00) vs. the model-calculated range of (102.49 to 106.33). In both cases the increased RBC flux (in comparison to the basis of 100 millimole of CO_2_ plus H_2_CO_3_ uptake) is needed to counteract the carbon loss due to the CO_2_ releasing reactions such as isocitrate dehydrogenase (ICD) and pyruvate dehydrogenase (PDH). We find that flux ranges, under the maximum biomass production assumption, of reactions such as glucose 6-phosphate dehydrogenase (G6PD), 6-phosphogluconolactonase (6PGL) and phosphogluconate dehydrogenase (6PGD) in oxidative pentose phosphate (OPP) pathway are negligible (0.00 to 0.03). In contrast, the experimentally derived range for OPP is (12 to 21). This is approximately equal to the difference between the model-predicted vs. experimentally deduced RBC reaction range implying the persistence of OPP flux even under the photoautotrophic condition [35] despite the presence of a more efficient NADPH production route through photosynthesis as predicted by the model (under max biomass). The high values Young et al. [35] obtained for the OPP fluxes were surprising as OPP is not a very efficient route for cyanobacteria to generate reducing power. This may reflect some inherent biological constraint that is not captured by the optimality assumption.

Model predicted lower flux ranges for RBC are propagated to seven other reactions in the Calvin cycle (i.e., phosphoglycerate kinase (PGK), glyceraldehyde-3-phosphate dehydrogenase (13PDG), triose-phosphate isomerase (TPI), transketolase (TKT1), ribose-5-phosphate isomerase (RPI), ribulose 5-phosphate 3-epimerase (RPE) and phosphoribulokinase (PRK). The remaining six reaction fluxes with lower model predicted fluxes compared to measurements [35] are all in the TCA cycle (i.e., citrate synthase (CS), aconitase (ACONT), isocitrate dehydrogenase (ICD), succinate dehydrogenase (SUCD) and malic enzyme (ME1 and ME2) reactions). Even under the max biomass assumption, SUCD is not required to carry any flux due to the presence of other succinate dehydrogenases (as part of respiratory chain) in the *i*Syn731 model. Furthermore, in contrast with experimental observations, under the maximum biomass assumption, the model predicts no flux through the malic enzyme (ME) reactions presumably because it is a less energy-efficient route (i.e., phosphoenolpyruvate → oxaloacetate → malate → pyruvate) for pyruvate generation than the pyruvate kinase (PYK) reaction [35].

There are nine reactions with experimentally derived ranges completely subsumed within the ones derived under the maximum biomass assumption. Five of them are in the Calvin cycle (i.e., fructose-bisphosphate aldolase (FBA), fructose-bisphosphatase (FBP), Sedoheptulose 1,7-bisphosphate D-glyceraldehyde-3-phosphate-lyase (SBGPL), sedoheptulose-bisphosphatase (SBP) and bidirectional transaldolase (TAL)). The first four reactions are essential with experimentally deduced flux ranges of (53.00 to 66.00) for FBA and FBP and (29.00 to 43.00) for SBGPL and SBP. In contrast, the calculated flux ranges (−0.08 to 73.17) for FBA and SBGPL and (0.00 to 73.17) for FBP and SBP imply that they are *in silico* non-essential. As depicted in Figure 1, these reactions are involved in the production of sedoheptulose 7-phosphate (S7P) from fructose 1,6-bisphosphate (FDP). An alternative production route for S7P is afforded in the model through the bidirectional transaldolase (TAL) reaction from fructose 6-phosphate (F6P) alluding to an explanation for the wider flux ranges derived using the model. Experimental and model predicted flux ranges for TAL are (−6.00 to 9.00) and (−35.93 and 37.32), respectively. Upon restricting the TAL flux ranges in the calculations to the ones found experimentally, the flux variability analysis shrinks the flux ranges for FBA and FBP to (28.22 to 43.27) and (28.22 to 43.33) and for SBGPL and SBP to (29.82 to 44.87) and (29.82 to 44.87), respectively which are very close to the experimentally measured ranges. This is indicative that in addition to the maximization of biomass formation, additional restrictions (e.g., photosynthetic efficiency and relative selectivity of RuBISCO for carboxylation over oxidation) limit the range of fluxes that the aforementioned glycolytic fluxes may span *in vivo*. Note that the presence of experimentally measured fluxes is important to test the model and the adopted maximization principle. We were fortunate in this case to have access to such data as for most organisms they are absent.

Phosphoglycerate mutase (PGM) and enolase (ENO) reactions have very similar model derived and experimentally obtained flux ranges. Model-predicted flux values of the remaining two reactions, pyruvate kinase (PYK) and pyruvate dehydrogenase (PDH), could reach as low as zero due to the metabolic flexibility that the *i*Syn731 model possesses by having alternate enzymes with different cofactor specificities. The max biomass flux range of fumarase (FUM) is found to be (−7.26 to 1.49), compared to the experimentally measured (1.70 to 2.00). Therefore, it appears that under the photoautotrophic condition, the forward direction is kinetically favorable. By restricting the reaction to be irreversible the model predicted a FUM flux range of (0.00 to 1.49) which is close to the experimentally derived one (see Figure 1). However, contrary to MFA measurements these reactions (FUM and ME) are dispensable for *in silico* biomass production.

### *i*Syn731 Model Testing Using *in vivo* Gene Essentiality Data

The quality of model *i*Syn731 for Synechocystis 6803 was tested using experimental data on the viability (or lack thereof) of single gene knockouts. We used the CyanoMutants database [34], [42] that includes *in vivo* gene essentiality data for 119 genes (i.e., 19 essential and 100 nonessential) with metabolic functions in *i*Syn731 model. Cases that were flagged with incomplete segregation in the database were omitted in *i*Syn731 model comparisons. We examined the feasibility of biomass production for the model *i*Syn731 by comparing the maximum biomass formation upon imposing the gene knockout with the maximum theoretical yield of the *wild-type* organism. A threshold of 10% of the maximum theoretical yield was used as a cutoff [43]. Comparisons between *in vivo* and *in silico* results led to four possible outcomes, as previously delineated by Kumar *et al*., GG, GNG, NGG and NGNG [43]. Initially, the model correctly predicted 18 out of 19 essential genes (i.e., 18 NGNG and 1 GNG) and 74 out of 100 non-essential genes (i.e., 73 GG and 27 NGG). We next explored the causes of these discrepancies and attempted to mitigate them whenever possible.

The single GNG case corresponds to mutant Δ*chlA_I_* exhibiting no growth under aerobic conditions [44]. The ChlA_I_ system is a Mg-protoporphyrin IX monomethylester (MPE) cyclase system that is responsible for forming the isocyclic ring (E-ring) in chlorophylls under aerobic conditions [44]. The model allowed for the BchE and ChlA_II_ systems (alternate cyclase systems) to complement for the loss of the ChlA_I_ system leading to an *in silico* viable mutant. However, the same literature source [44] suggested that both BchE and ChlA_II_ systems are unlikely to be active under aerobic conditions and thus rescue mutant Δ*chlA_I_*. This prompted the introduction of a regulatory restriction in *i*Syn731 model where only ChlA_I_ reactions were active under aerobic conditions as MPE while ChlA_II_ and BchE system reactions were deactivated. Using these regulatory restrictions resolves the single GNG inconsistency.

Twenty (out of 27) NGG cases were associated with Photosystem I (PSI), Photosystem II (PSII) and other photosynthesis reactions. While reconstructing the model, we assumed that all genes involved in photosynthetic reaction system were essential to the functioning of the overall system. Published literature [45]–[49] suggests that genes involved in photosynthetic reactions form complex interdependencies. We used NCBI COBALT multiple sequence alignment tool [50] to construct a phylogenetic tree of the genes associated with each photosystem along with BLASTp searches to identify putative complementation relationships between genes to explain the inconsistencies between the predicted *in silico* and *in vivo* growth. Genes deemed homologous (i.e., lie adjacent in the phylogenetic tree) were linked with “OR” GPR relations implying that the loss of one gene can be complemented by the other. Seven out of twenty NGG cases (i.e., *psaD, psaI* and *psbA2* for PSI and PSII and *cpcC2, cpcC1, cpcD,* and *apcD* for other photosynthesis reactions) were resolved by modifying the corresponding GPR using an OR relation [46], [47], [51]–[54]. However, no phylogenetically adjacent or related (or homologous) genes were found for the remaining 13 NGG cases (*psaE, psbD2, psbO, psbU, psbV, psb28, psbX, psb27, petE, cpcA, cpcB, apcE, apcF*) [48], [49], [51], [54]–[58]. For these cases, the genes were deemed nonessential to the functioning of the reactions in question (i.e., photosynthesis reactions) and thereby the corresponding GPRs were modified to show an OR relation between each of these genes and an ‘*unknown gene’*, similar to what was previously performed in the refinement of the *i*MM904 model [59] (see File S5 for detailed information).

The remaining seven NGG cases are associated with a variety of metabolic functions. One such case is the Δ*modBC* mutant corresponding to the sole ABC molybdate transporter in the model. Literature evidence [60] revealed that a related cyanobacterium, *Anabaena variabilis* ATCC 29413, could continue to grow despite the loss of its molybdate ABC transporter due to the presence of another low affinity molybdate transporter or an inducible sulfate transport system that can serve as a low affinity molybdate transporter when required. We found the same gene coding for the sulfate transporter in *A. variabilis* (*cysA*) in the *i*Syn731 model allowing the resolution of the discrepancy by adding a *cysA*-linked alternate molybdate transporter. Another NGG case is mutant Δ*crtO* that cannot produce echinenone (a biomass component) in *i*Syn731 with no effect on observed growth. Therefore, it appears that *i*Syn731 cannot capture the flexibility of *Synechocystis* 6803 metabolism [61] when echinenone production is restricted. The remaining five NGG cases are spread across many metabolic pathways. The Δ*ctaA* mutant eliminates the copper ABC transporter without affecting growth, which alludes to the existence of another unknown mode of copper uptake not present in *i*Syn731 [62], [63]. The Δ*menG* mutant eliminates a reaction for the production of phylloquinone while mutant Δ*ppd* affects the production of homogentisate, a precursor for both tocopherols and plastoquinone. Finally, the Δ*vte3* mutant affects the production of both plastoquinone and α-tocopherol [64] and the viable Δ*ccmA* mutant restricts the production of chorismate (a precursor to aromatic amino acids) and also restricts carboxysome formation [65]–[67]. These six inconsistencies between the model predictions and growth data imply that the cyanobacterium can co-opt another metabolic process to (partially) complement for the gene loss. Unlike the case of the Δ*modBC* mutant, we have found no plausible mechanism for the six remaining mutants.

**Figure 2:**
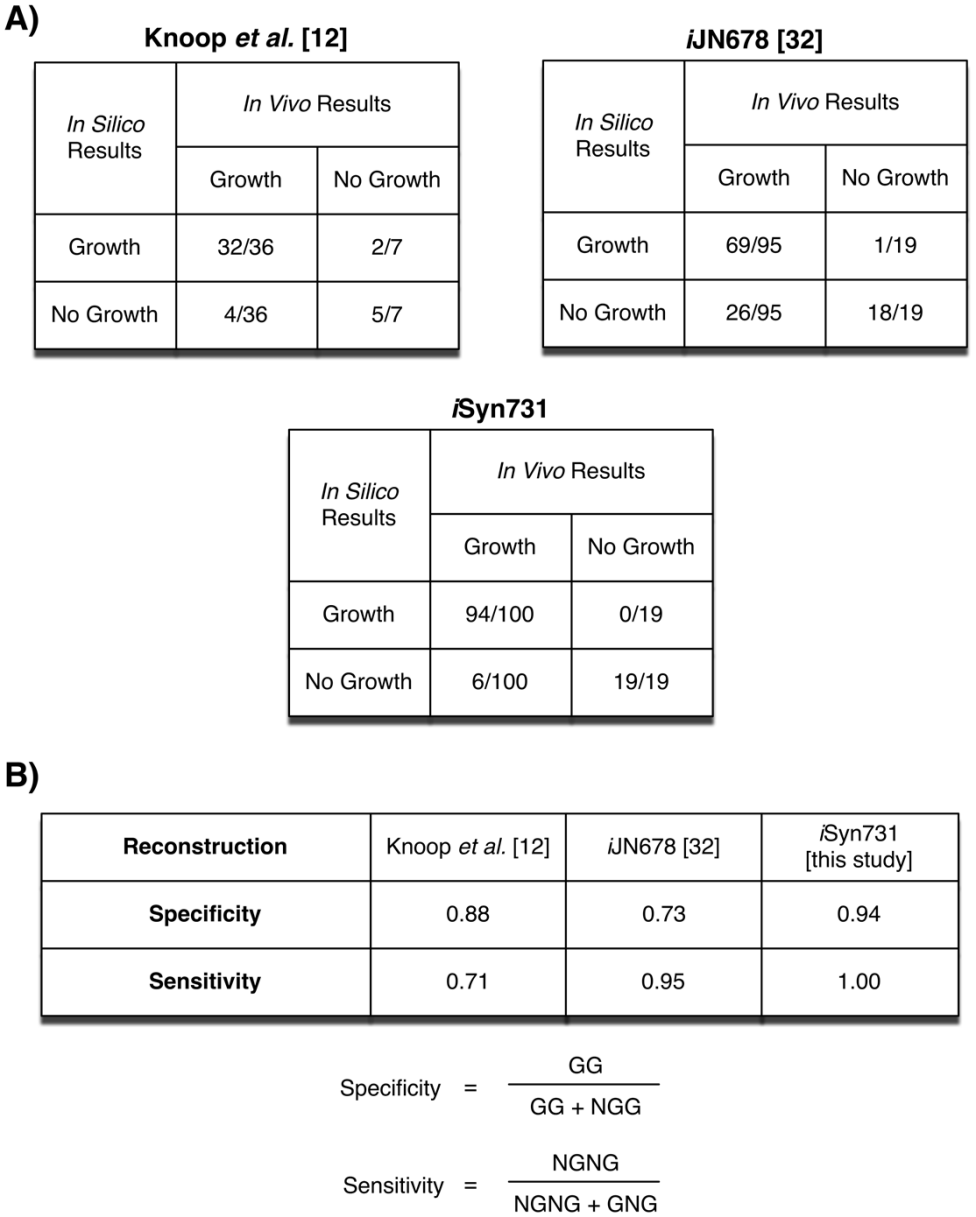
Comparison of gene essentiality/viability data with predictions by a number of *Synechocystis* 6803 models. (A) Tabulated growth (i.e., G) or non-growth (i.e., NG) predictions and experimental data. The first number denotes the number of GG, GNG, NGG and NGNG combinations whereas the second number signifies the number of experimentally observed lethal (or viable) mutants, and (B) Definition and comparison of specificity and sensitivity of all three models. Note that GG denotes both *in silico* and *in vivo* growth, NGG represents no growth *in silico* but *in vivo* growth. NGNG implies no growth for either *in silico* or *in vivo*, whereas GNG marks growth *in silico* but no growth *in vivo*.

After resolving the discrepancies, as described above, *i*Syn731 correctly predicted all 19 essential genes (i.e., 19 NGNG and 0 GNG) and 94 (out of 100) non-essential genes (i.e., 94 GG and 6 NGG). Figure 2 shows our results and comparisons against two other available *Synechocystis* 6803 models by Knoop *et al*. [12] and Nogales *et al*. [32]. We used the CyanoMutants database [34] to identify 114 genes (i.e., 19 essential and 95 nonessential) having metabolic functions in the *i*JN678 model by Nogales *et al*. [32]. Out of 114 genes the *i*JN678 model correctly predicted 18 essential genes (i.e., 18 NGNG and 1 GNG) and 69 non-essential genes (i.e., 69 GG and 26 NGG). The model by Knoop *et al.* [12] was tested for 51 mutants but we found that only 43 (i.e., 7 essential and 36 non-essential) of them were reported to have complete segregation [34]. Of these 43, Knoop *et al.*’s [12] model correctly predicted 5 essential genes (i.e., 5 NGNG and 2 GNG) and 32 nonessential genes (i.e., 32 GG and 4 NGG). The specificity and sensitivity of each of these three models were also calculated and displayed at the bottom of Figure 2.

All 114 genes tested for *i*JN678 were also present in the *i*Syn731 model. 26 NGG and one GNG cases present in *i*JN678 model correspond to NGG and GNG cases that were either fixed or still present in *i*Syn731 as discussed before. Lethal mutant Δ*ppa* is correctly predicted as NGNG in *i*Syn731 but deemed GNG in Knoop *et al.* [12] model. This was because *ppa* in *i*Syn731 codes for the degradation of both triphosphate into diphosphate and diphosphate to phosphate. Only the latter activity is linked to *ppa* in the Knoop et al’s model. Out of 4 NGG cases in [12], two involve Δ*cmpA* and Δ*cmpB* mutants. Both these genes are involved in the ABC transporter system for bicarbonate from periplasm to cytosol. *i*Syn731 avoids this inconsistency as it contains an alternate sodium and bicarbonate co-transport system.

### Model Comparisons

#### *Synechocystis* 6803 model comparisons

**Figure 3:**
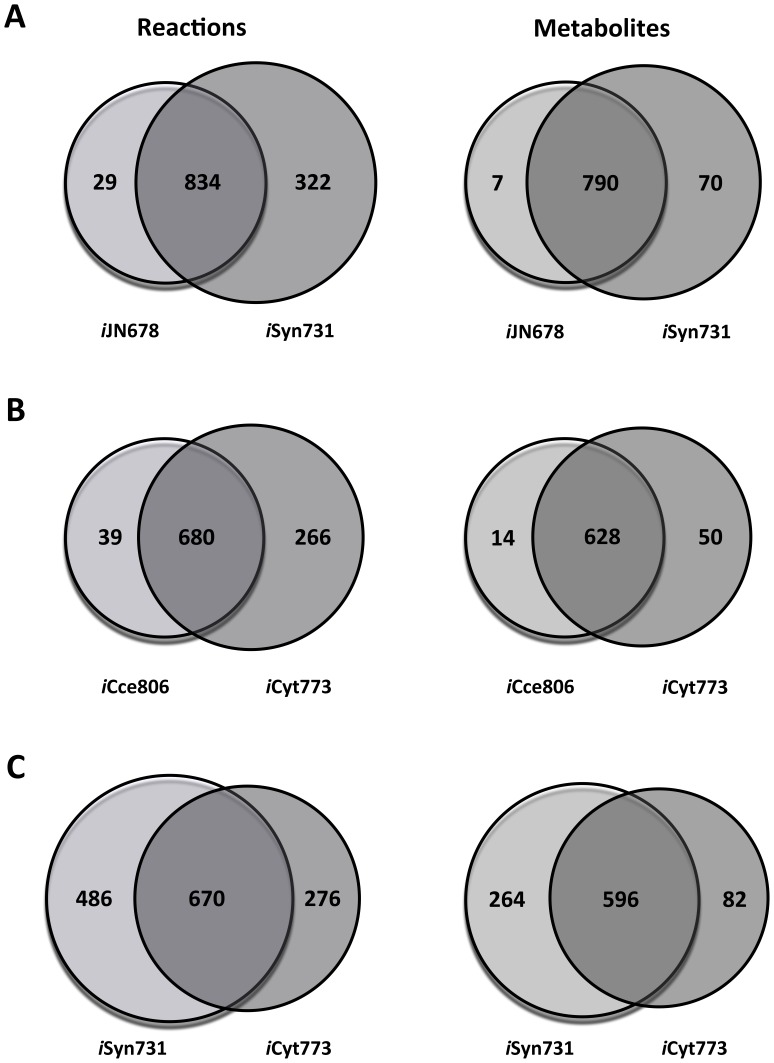
Venn diagram depicting (common and unique) reactions and metabolites between (A) *i*JN678 [**32**] and *i*Syn731, (B) *i*Cce806 [**25**] and *i*Cyt773, and (C) *i*Syn731 and *i*Cyt773 models.

**Figure 4:**
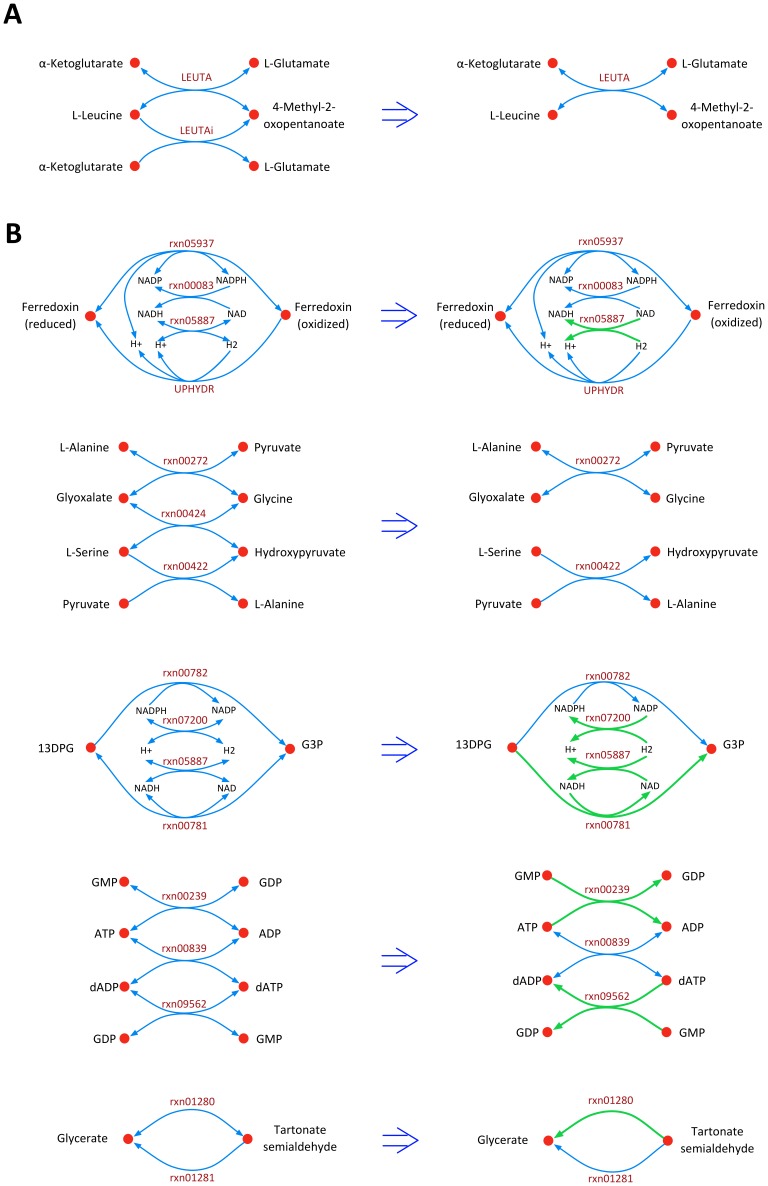
Schematics that illustrate the thermodynamically infeasible cycles and subsequent resolution strategies. (A) Cycles present in *i*JN678 [32], and (B) Cycles present in *i*Cce805 [25]. Blue colored lines represent the original reaction directionality whereas green ones denote modified directionality to eliminate cycle.

The *i*Syn731 model integrates the description in the photosystems of the model presented by Nogales et al. [32] and adds additional detail. One notable difference is that *i*Syn731 uses a separate photon for each reaction center (i.e., PSI and PSII) as they are optimized for different ranges of wavelength [68], whereas *i*JN678 [32] uses a single photon shared by both photosystem reactions. As many as 322 new reactions (see Figure 3A), are added in *i*Syn731 distributed across many pathways. Most of the additions are in the lipid and fatty acid metabolism to support the synthesis of measured fatty acids and lipids present in the biomass equation. This list includes myristic acid (14-carbon saturated fatty acid) and lauric acid (12-carbon saturated fatty acid). *i*JN678 [32] contained four reactions exhibiting unbounded flux (i.e., two duplicate glycine cleavage reactions and two duplicate leucine transaminase reactions). They form a thermodynamically infeasible cycle (see Figure 4A for leucine transaminase reactions) that was resolved in *i*Syn731 by eliminating redundant functions. In addition, the glycine cleavage system was recast in detail by abstracting the separate action of the four enzymes (named the T-, P-, L-, and H-proteins) that ultimately catalyze the demethylamination of glycine.

**Figure 5:**
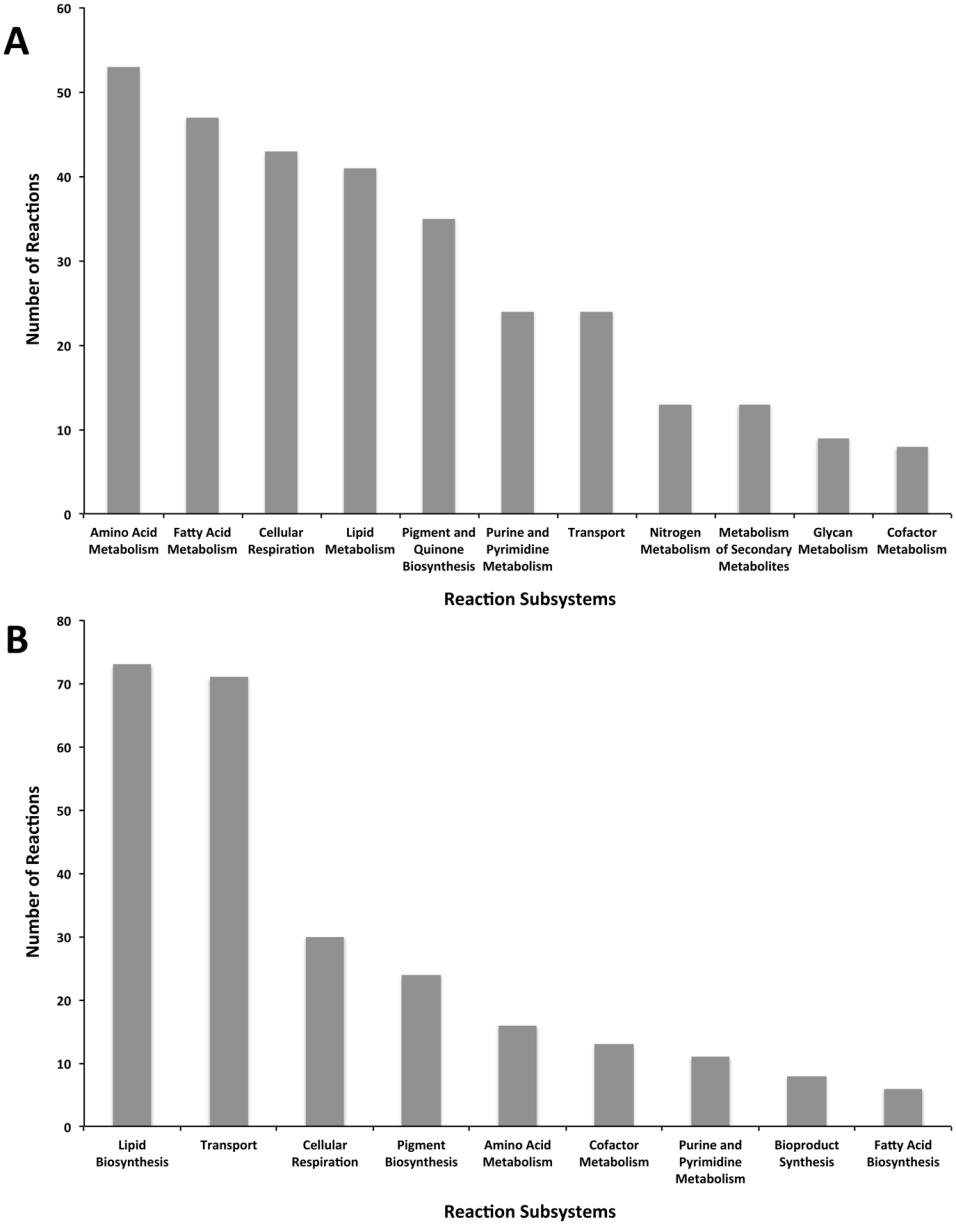
List of added reactions across pathways. (A) *i*Syn731 compared to *i*JN678 [32], and (B) *i*Cyt773 compared to *i*Cce806 [25].

*i*Syn731 improves upon *i*JN678 [32] by eliminating lumped reactions whenever a multi-step description is available and expands the range of functions carried out with alternate cofactors. As many as twelve reactions with an enoyl-[acyl-carrier-protein] reductase function were linked with not only NADP but also with the more rare NAD cofactor specificity. Another important difference between *i*Syn731 and *i*JN678 [32] is the cellular location of the CO_2_ fixation (i.e., ribulose-1,5-bisphosphate carboxylase/oxygenase (RuBisCO) enzyme). Literature [69], [70] shows that cyanobacteria possess a micro-compartment (i.e., carboxysome) encapsulating RuBisCO and carbonic anhydrase (CA) enzymes. *i*Syn731 adds carboxysome as a cellular compartment and also all necessary transport reactions [69], [70]. Recently, Zhang and Bryant [33] hypothesized the existence of a functional TCA cycle in most cyanobacterial species using a 2-ketoglutarate to succinate bypassing step. *i*Syn731 allows for a complete TCA cycle using the bypassing step. In addition, *i*Syn731 contains an intact heptadecane biosynthesis pathway as recently described [21] unlike earlier *Synechocystis* 6803 models [12], [29], [30], [32] (see Figure 5A for distribution of unique reactions in *i*Syn731).

#### *Cyanothece* 51142 model comparisons

The *i*Cyt773 model for *Cyanothece* 51142 improves upon the *i*Cce806 model [25]. *i*Cyt773 segregates reactions into the periplasm, thylakoid lumen, carboxysome, and cytoplasm compartments thus introducing an additional 60 transport reactions compared to *i*Cce806 [25]. Unlike *i*Cce806 [25], *i*Cyt773 does not track macromolecule synthesis for DNA, RNA, and proteins to maintain consistency with the *Synechocystis* 6803 model. This difference accounts for 69 genes present in *i*Cce806 [25] but absent from *i*Cyt773. *i*Cce806 [25] contained 15 reactions which formed five cycles that could carry unbounded metabolic flux (i.e., thermodynamically infeasible cycles). All these cycles were eliminated by restricting reaction directionality and eliminating reactions that were linear combinations of others (coded by the same gene) (see Figure 4B).

*i*Cyt773 contains 43 unique genes and 266 unique reactions (including transport and alternate cofactor utilizing reactions) as shown in Figure 3B. Figure 5B depicts the distribution of the new reactions across different pathways. Most of the additions are found in lipid and pigment biosynthesis pathways. The *i*Cyt773 model captures in detail the lipid biosynthesis pathway composed of 73 reactions and links as many as 28 biomass precursor lipids (e.g., sulfoquinovosyldiacylglycerols, monogalactosyldiacylglycerols, digalactosyldiacyl-glycerols, and phosphatidylglycerols) directly to the biomass equation. The porphyrin and chlorophyll metabolism and carotenoid biosynthesis pathways were updated to include 24 reactions for the production of accessory pigments such as echinenone, an accessory pigment, and (3Z)-phycocyanobilin, a phycobilin. Accessory pigments donate electrons to chlorophyll rather than directly to photosynthesis. Phycobilins are adapted for many wavelengths not absorbed by chlorophyll thus broadening the spectrum useful for photosynthesis. The variety of pigments in cyanobacteria is well documented [71]–[73] providing so far untapped avenues for engineering increased efficiency in photosynthesis and control of electron transfer processes in biological systems. Another new function in *i*Cyt773 is L-Aspartate Oxidase. L-Aspartate Oxidase allows the deamination of aspartate, forming oxaloacetate a key TCA-cycle metabolite and ammonia. The impact of this addition to *i*Cyt773 is not evident under the photoautotrophic condition but becomes relevant for growth in a medium containing aspartate. *i*Cyt773 also uniquely supports the synthesis of pentadecane as documented by Schirmer et al. [21] and contains an (almost) complete non-fermentative citramalate pathway as suggested by Wu et al. [22].

A number of lumped reactions in *i*Cce806 [25] were recast in detail. For example, pyruvate dehydrogenase (PDH) is a three-enzyme complex that carries out the biotransformation of pyruvate to acetyl-CoA in three steps using five separate cofactors (i.e., TPP, CoA, FAD, lipoate, and NAD). Similar detail was used for lumped steps in the metabolism of glycine, histidine, and serine. All additions to the list of reactions in *i*Cyt773 were corroborated using genome annotations [9] or published literature [20]–[22], [74] with the exception of ten enzymes, whose function in the lipid and pigment biosynthesis pathways was required for biomass production.

A shift in biomass composition was observed under light, dark, and nitrate supplemented (light and dark) conditions. These differences were captured in four separate biomass descriptions present in *i*Cyt773. In addition, we used data from Stockel et al. [75] on the diurnal oscillations for approximately 20% of proteins in *Cyanothece* 51142 to identify regulatory reaction shutdowns in our metabolic model. File S4 lists the reactions that were inactivated under light and dark conditions, respectively. As expected, the nitrogenase genes *cce_0559* and *cce_0560*, known to be active in the absence of light, exhibited low spectral counts under light conditions. In contrast, photosystem II gene *cce_1526*, showed no spectral count under dark conditions. Unexpectedly, the data suggested that the Mehler reactions associated gene (*cce_2580*), known to be active in *Synechocystis* 6803 [76] and expected to be active in *Cyanothece* 51142, exhibited lower expression in light than in dark conditions.

#### *i*Syn731 and *i*Cyt773 models comparison

Figure 3C illustrates the total number of common and unique reactions and metabolites between *i*Syn731 and *i*Cyt773 models. The *Cyanothece* 51142 genome [9], [77] is 1.5 times larger than the one for *Synechocystis* 6803 [11], nevertheless *i*Cyt773 is smaller than *i*Syn731 due to differences in the level of detail of annotation and biochemical characterization. As many as 670 reactions and 596 metabolites are shared by both models corresponding to 47% and 63% of the total reactome and metabolome, respectively (see Figure 3C). The higher degree of conservation of metabolites (as opposed to reactions) across the two cyanobacteria suggests that lifestyle adaptations tend to usher new enzymatic activities that most of the time make use of the same metabolite pool without introducing new metabolites. There are 486 reactions that are unique to *i*Syn731 with no counterpart in *i*Cyt773. These reactions are not preferentially allotted to a handful of specific pathways. Instead they are spread over tens of different pathways. Primary metabolism reactions dispersed throughout fatty acid biosynthesis, lipid metabolism, oxidative phosphorylation, purine and pyrimidine metabolism, transport and exchange reactions account for 295 reactions. Secondary metabolism including chlorophyll and cyanophycin metabolism, folate, terpenoid, phenylpropanoid and flavonoid biosynthesis accounts for the remaining 191 *i*Syn731-specific reactions. Interestingly, the 276 iCyt773-specific reactions span the same set of diverse pathways implying that the two organisms have adopted unique/divergent biosynthetic capabilities for similar metabolic needs. Fifty-eight span primary metabolism pathways such as purine and pyrimidine metabolism, fatty acid and lipid biosynthesis, amino acid biosynthesis. The remaining 218 reactions describe secondary metabolism such as terpenoid biosynthesis, chlorophyll and cyanophycin biosynthesis, plastoquinone and phyloquinone biosynthesis (see File S6 for detail information). The much larger set of unique *i*Syn731-specific reactions compared to *i*Cyt773 reflect more complete genome annotation and biochemical characterization rather than augmented metabolic versatility.

**Figure 6:**
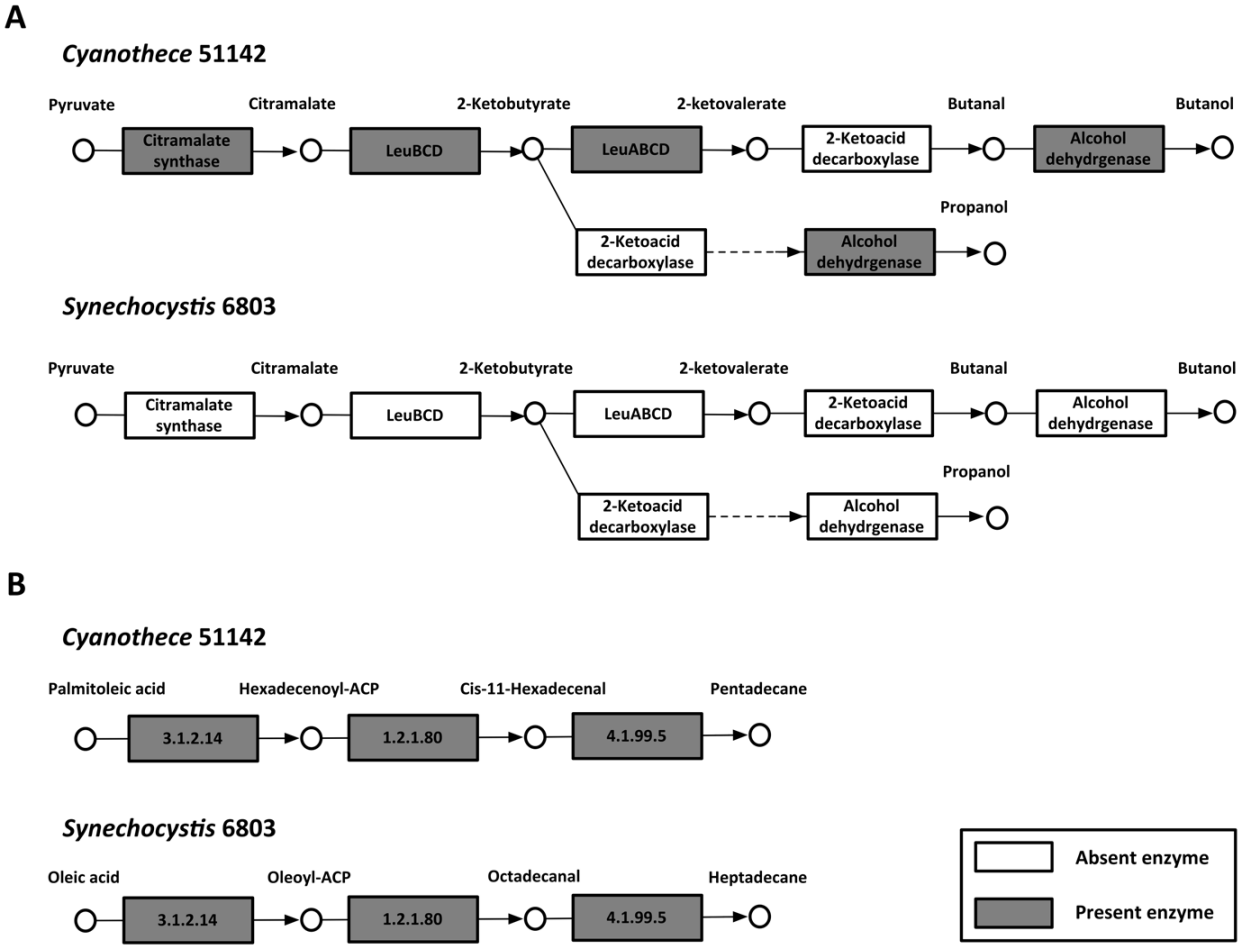
Examples of pathways that differ between the two cyanobacteria. (A) Nonfermentative alcohol production pathway highlighting the present and absent enzymes in *Cyanothece* 51142 and *Synechocystis* 6803, and (B) Alkane biosynthesis pathways in *Cyanothece* 51142 and *Synechocystis* 6803.

A number of distinct differences in metabolism between the two organisms have been accounted for in the two models. For example, *i*Cyt773 does not have the enzyme threonine ammonia-lyase, which catalyzes the conversion of threonine to 2-ketobutyrate and as a consequence lacks the traditional route for isoleucine synthesis. Instead it employs part of the alternative citramalate pathway for isoleucine synthesis with pyruvate and acetyl-CoA as precursors. Follow up literature queries revealed the existence of this alternative pathway in *Cyanothece* 51142 [22]. Ketobutyrate, an intermediate in the citramalate pathway, can be readily converted to higher alcohols, such as propanol and butanol, via a non-fermentative alcohol production pathway. Using the *i*Cyt773 model, we determined that only 2-ketoacid decarboxylase is missing from these three-step processes. In contrast, *i*Syn731 was found to have only the traditional route for isoleucine production with the citramalate pathway completely absent (see Figure 6A). In another example, the fermentative 1-butanol pathway is known to be incomplete in both organisms. By querying the developed models we can pinpoint exactly which steps are absent. Specifically, the conversion between 3-hydroxybutanoyl-CoA and butanal is missing in both models. In addition to higher alcohols, higher alkanes (C13 and above) are important biofuel molecules as the main constituents of diesel and jet fuel [21]. Recently reported [21] novel genes involved in the biosynthesis of alkanes in several cyanobacterial strains were incorporated in the models. Metabolic differences in *Cyanothece* 51142 and *Synechocystis* 6803 lead to the production of different alkanes (e.g., pentadecane in *Cyanothece* 51142 and heptadecane in *Synechocystis* 6803) (see Figure 6B).

Model *i*Cyt773, in contrast to *i*Syn731, does not have a complete urea cycle as it lacks the enzyme L-arginine aminohydrolase catalyzing the production of urea from L-arginine. Literature sources [77], [78] support this finding and explain the absence of a functional urea cycle as a consequence of the nitrogen-fixation ability of *Cyanothece* 51142 [79], [80]. Because *Cyanothece* 51142 can fix nitrogen directly from the atmosphere and produce ammonium via the enzyme nitrogenase, genes corresponding to the activity of L-arginine aminohydrolase and urease (for breaking down urea) become redundant, explaining why they are not present in its genome [80]. In addition to nitrogen metabolism, *i*Cyt773 and *i*Syn731 models reveal marked differences in anaerobic metabolic capabilities. Unlike *i*Syn731, *i*Cyt773 includes a L-lactate dehydrogenase activity that enables the complete fermentative lactate production pathway. On the other hand, *i*Syn731 contains the anaerobic chlorophyll biosynthetic pathway using enzyme protoporphyrin IX cyclase (BchE) that is absent in *i*Cyt773. Other differences in metabolism include lipid and fatty acid synthesis, fructose-6-phosphate shunt and nitrogen fixation. Model *i*Syn731 traces the location of the double bond for unsaturated fatty acid synthesis pathways, as two separate isomers of unsaturated C_18_ fatty acids are part of the biomass description. *i*Cyt773 allows for the shunting of fructose-6-phosphate into erythrose-4-phosphate along with acetate and ATP using the fructose-6-phosphate phosphoketolase activity. Finally, both *i*Syn731 and *i*Cyt773 contain multiple hydrogenases allowing both to produce hydrogen. However, only the latter has a nitrogenase activity that can fix nitrogen while simultaneously producing hydrogen.

### Using *i*Syn731 and *i*Cyt773 to Estimate Production Yields

We tested the recently developed models *i*Syn731 and *i*Cyt773 by comparing the predicted maximum theoretical product yields with experimentally measured values for two very different metabolic products: isoprene and hydrogen. Isoprene, a volatile hydrocarbon and potential feedstock for biofuel, is mostly produced in plants under heat stress [13]. Cyanobacteria offer promising production alternatives as they can grow to high densities in bioreactors and produce isoprene directly from photosynthesis intermediates [13]. It was reported [13] that *Synechocystis* 6803 has all but one gene (encoding isoprene synthase) in the methyl-erythritol-4-phosphate (MEP) pathway for isoprene synthesis from dimethylallyl phosphate (DMAPP). Upon cloning the isoprene synthase from kudzu vine (*Pueraria montana*) into *Synechocystis* 6803 isoprene production was demonstrated using sunlight and atmospheric CO_2_ of 4.3×10^−4^ mole isoprene/mole carbon fixed [81]. We calculated the maximum isoprene yield using *i*Syn731 to be 3.63×10^−5^ mole isoprene/mole carbon fixed upon adding the isoprene synthase activity to the model and simulating the conditions described in [41] under maximum biomass production. Similar isoprene yields were obtained with *i*JN678 [32] while earlier models of *Synechocystis* 6803 [12], [29]–[31] lack the MEP pathway (partially or completely) and thus do not support isoprene production. The underestimation of the experimentally observed isoprene yield by the model predicted maximum yield may be due to sub-optimal growth of the production strain, differences in the list of measured biomass components, missing isoprene-relevant reactions from the model or more likely a combination of the above factors.

Both *Cyanothece* 51142 and *Synechocystis* 6803 produce hydrogen by utilizing nitrogenase and hydrogenase activities, respectively [19]. Under subjective dark conditions [19] whereby (i) stored glycogen acts as a carbon source, (ii) photosynthesis harnesses light energy, and (iii) nitrogenase activity is not restricted, hydrogen production yield for *Cyanothece* 51142 was measured at 49.67 mole/mole glycogen consumed. Simulating the same conditions in *i*Cyt773 and *i*Cce806 [25] leads to maximum theoretical yields for hydrogen production of 48.43 mole/mole glycogen and 102.4 mole/mole glycogen, respectively. The entire amount of hydrogen produced in *i*Cyt773 is due to the nitrogenase activity. In contrast, the predicted doubling of the maximum hydrogen yield in *i*Cce806 is due to the utilization of the reverse direction of two hydrogen dehydrogenase reactions without any nitrogenase activity. Utilization of the nitrogenase reaction requires the use and recycling of more ATP than simply running the dehydrogenase reactions in reverse. However, it has been reported that hydrogen production in *Cyanothece* 51142 is primarily mediated by the nitrogenase enzyme [19] in the dark phase. This lends support to the irreversibility of the dehydrogenase reactions (under dark condition) as present in the *i*Cyt773 model. Experimental results for *Synechocystis* 6803 support up to 4.24 mole/mole glycogen consumed [19], [82] of hydrogen production. *i*Syn731 predicts a maximum hydrogen theoretical yield of 2.28 mole/mole glycogen consumed while *i*JN678 [32] yields a value of 2.00 mole/mole glycogen consumed. Again the factors outlined for isoprene production may explain the lower theoretical yields predicted by the two models. The small difference between the model predicted yields is due to the presence of one step lumped biotransformation between isocitrate and oxoglutarate via isocitrate dehydrogenase in *i*JN678 [32]. *i*Syn731 describes this biotransformation in two steps (isocitrate → oxalosuccinate → oxoglutarate) [83] generating an additional NADPH and subsequently more hydrogen via the hydrogenase reaction.

### Conclusion

In this paper, we expanded upon existing models to develop two genome-scale metabolic models (*Synechocystis i*Syn731 and *Cyanothece i*Cyt773) for cyanobacterial metabolism by integrating all available knowledge available from public databases and published literature. All metabolite and reaction naming conventions are consistent between the two models allowing for direct comparisons. Systematic gap filling analyses led to the bridging of a number of network gaps in the two models and the elimination of orphan metabolites. Two separate biomass equations as well as two different versions of *Cyanothece i*Cyt773 models were developed for light and dark phases to represent diurnal regulation. The development of two separate models for *Cyanothece* 51142 (i.e., light and dark) provides the two “end-points” for the future development of dynamic metabolic models capturing the temporal evolution [36], [84]–[86] of fluxes during the transition phases DFBA [87]. Comparisons against available ^13^C MFA measurements for *Synechocystis* 6803 [35] revealed that the *i*Syn731 model upon biomass maximization yields flux ranges that are generally consistent with experimental data. Discrepancies between the two identify metabolic nodes where regulatory constraints are needed in addition to biomass maximization to recapitulate physiological behavior. The ability of *i*Syn731 to predict the fate of single gene knock-outs was further improved (specificity of 0.94 and sensitivity of 1.00) by reconciling *in silico* growth predictions with *in vivo* gene essentiality data [34]. Similar analyses could also be carried out for *Cyanothece i*Cyt773 model once such flux measurements and *in vivo* gene essentiality data become available.

It is becoming widely accepted that focusing on a single pathway at a time without quantitatively assessing the system-wide implications of genetic manipulations may be responsible for suboptimal production levels. By accounting for both primary and some secondary metabolism pathways, the *Cyanothece i*Cyt773 model can be used to explore *in silico* the effect of genetic modifications aimed at increased production of useful biofuel molecules. By taking full inventory of *Cyanothece* 51142 metabolism (as abstracted in *i*Cyt773), and applying available strain optimization techniques [88], [89] optimal gene modifications could be pursued for a variety of targets in coordination with experimental techniques. In particular, the availability of a microaerobic environment in *Cyanothece* 51142 at certain times during the diurnal cycle can be exploited for the expression of novel pathways that are not usually found in oxygenic cyanobacterial strains that largely maintain an aerobic environment. However, the use of *Cyanothece* 51142 as a bio-production platform is currently hampered by the inability to efficiently carry out genetic modifications.

By systematically cataloguing the shared (and unique) metabolic content in *i*Syn731 and *i*Cyt773, successful genetic interventions assessed experimentally for *Synechocystis* 6803 can be “translated” to *Cyanothece* 51142. For example, it has been reported [90], [91] that overproduction of fatty alcohols can be achieved in *Synechocystis* 6803 upon cloning a fatty acyl-CoA reductase (*far*) from Jojoba (*Simmondsia chinensis*) and the over-expression of gene *slr1609* coding for an acyl-ACP synthetase. By using models *i*Syn731 and *i*Cyt773 we can infer that in addition to cloning *far* from Jojoba, over-expression of gene *cce_1133* coding for a native acyl-ACP synthetase would be needed to bring about the same overproduction in *Cyanothece* 51142.

## Supporting Information

File S1 *Synechocystis i*Syn731 model along with established GPR, metabolite, gene and protein information.
(XLSX)

File S2 *Cyanothece i*Cyt773 model along with established GPR, metabolite, gene and protein information.
(XLSX)

File S3 Biomass component measurements and stoichiometry of biomass equation.
(XLSX)

File S4 Reactions with diurnal activation/inactivation.
(XLSX)

File S5 Comparison of *in silico* vs. *in vivo* gene essentiality results for *i*Syn731 and modifications made in GPR associations.
(XLSX)

File S6 Comparison between *Synechocystis i*Syn731 and *i*Cyt773 models in terms of genes, proteins, reactions and metabolites.
(XLSX)

File S7 SBML file of *Synechocystis i*Syn731 model.
(XML)

File S8 SBML file of *Cyanothece* 51142 *i*Cyt773 model.
(XML)
